# Inequalities in physical comorbidity: a longitudinal comparative cohort study of people with severe mental illness in the UK

**DOI:** 10.1136/bmjopen-2015-009010

**Published:** 2015-12-15

**Authors:** Siobhan Reilly, Ivan Olier, Claire Planner, Tim Doran, David Reeves, Darren M Ashcroft, Linda Gask, Evangelos Kontopantelis

**Affiliations:** 1 Division of Health Research, Faculty of Health and Medicine, Lancaster University, Lancaster, UK; 2 Manchester Institute of Biotechnology, University of Manchester, Manchester, UK; 3 NIHR School for Primary Care Research, Centre for Primary Care, Institute of Population Health, University of Manchester, Manchester, UK; 4 Department of Health Sciences, University of York, York, UK; 5 Centre for Biostatistics, Institute of Population Health, University of Manchester, Manchester, UK; 6 Centre for Pharmacoepidemiology and Drug Safety, Manchester Pharmacy School, University of Manchester, Manchester, UK; 7 Centre for Health Informatics, Institute of Population Health, University of Manchester, Manchester, UK

**Keywords:** severe mental illness, comorbidity, EPIDEMIOLOGY, MENTAL HEALTH, physical health

## Abstract

**Objectives:**

Little is known about the prevalence of comorbidity rates in people with severe mental illness (SMI) in UK primary care. We calculated the prevalence of SMI by UK country, English region and deprivation quintile, antipsychotic and antidepressant medication prescription rates for people with SMI, and prevalence rates of common comorbidities in people with SMI compared with people without SMI.

**Design:**

Retrospective cohort study from 2000 to 2012.

**Setting:**

627 general practices contributing to the Clinical Practice Research Datalink, a UK primary care database.

**Participants:**

Each identified case (346 551) was matched for age, sex and general practice with 5 randomly selected control cases (1 732 755) with no diagnosis of SMI in each yearly time point.

**Outcome measures:**

Prevalence rates were calculated for 16 conditions.

**Results:**

SMI rates were highest in Scotland and in more deprived areas. Rates increased in England, Wales and Northern Ireland over time, with the largest increase in Northern Ireland (0.48% in 2000/2001 to 0.69% in 2011/2012). Annual prevalence rates of all conditions were higher in people with SMI compared with those without SMI. The discrepancy between the prevalence of those with and without SMI increased over time for most conditions. A greater increase in the mean number of additional conditions was observed in the SMI population over the study period (0.6 in 2000/2001 to 1.0 in 2011/2012) compared with those without SMI (0.5 in 2000/2001 to 0.6 in 2011/2012). For both groups, most conditions were more prevalent in more deprived areas, whereas for the SMI group conditions such as hypothyroidism, chronic kidney disease and cancer were more prevalent in more affluent areas.

**Conclusions:**

Our findings highlight the health inequalities faced by people with SMI. The provision of appropriate timely health prevention, promotion and monitoring activities to reduce these health inequalities are needed, especially in deprived areas.

**Strengths and limitations of this study**

- Large UK longitudinal study to explore the prevalence of severe mental illness (SMI) and comorbidity in the context of deprivation covering 12 years (2000–2012).
- Differences in the prevalence of SMI over time, between countries in the UK and regions in England are explored; increases observed between 2000 and 2012 and highest in areas of high deprivation.
- This research highlights the rising inequalities in the pattern and number of different comorbidities of this group of patients, a variable pattern of comorbidity across the different SMI subgroups and areas of high and low deprivation and has the potential to inform the provision of appropriate and timely health prevention, promotion and monitoring activities.
- Routinely collected clinical data were used, so we were not able to match controls on other important parameters such as obesity, unemployment, ethnicity, smoking status, alcohol or illegal drug use which may not be recorded.

## Introduction

It is well established that the physical health of people with severe mental illness (SMI) is much poorer than for people without SMI and that the causes of poor physical health in people with a SMI are complex and interactive.^1^ The factors that account for this include adverse effects of antipsychotic medication^2^ and unhealthy lifestyle behaviours which increase the likelihood of developing obesity, hypercholesterolaemia and metabolic syndrome, which in turn increase the risk of chronic diseases such as diabetes mellitus. Other barriers to recognising and managing physical conditions include difficulty in understanding healthcare advice, reduced motivation to adopt new lifestyle changes, poor treatment compliance, cognitive deficits, reduced pain sensitivity (induced by antipsychotic medication), poor communication and social skills.^3^ ^4^ Higher risk of comorbidity is often compounded by problems of engagement with the National Health Service (NHS) healthcare system, for example, reluctance of general practitioners (GPs) to participate in care,^5^ ^6^ which is reflected in the likelihood of patients being opted out (appropriately or not) but also refusing treatment.^5^ ^7^ ^8^ There is some evidence that health prevention and promotion activities in primary care are less frequent for people with SMI despite frequent contact with the system.^9^ As a result, premature mortality is much higher in people with SMI compared with those without SMI.^10^ ^11^ A number of both system and individual actions are necessary to address gaps in the treatment of physical health in people with SMI.^4^

Several cross-sectional studies have indicated that individuals with SMI have increased rates of physical illness compared with the general population;^2^ ^12–16^ however, these studies refer to data for limited periods of time, focus on Scotland or London. We extend this work by describing both SMI rates and patterns of comorbidity in a matched UK population over a 12-year period. A better understanding of comorbidity for people with SMI is necessary for improving estimates to inform policy and planning services.^17^ This paper examines the:

1. Prevalence of SMI in the UK by country (England, Northern Ireland (NI), Scotland and Wales), each region in England and each deprivation quintile in the UK, during the period 2000–2012.
2. Prevalence rates of 16 comorbidities in people with SMI during (1) 2000–2012 and (2) 2011/2012 for different types of SMI diagnoses (schizophrenia, bipolar disorder, affective disorder and other types of psychosis), compared with people without SMI and by the most affluent and most deprived quintile and (3) the 5-year period 2007/2008–2011/2012 for the combinations of comorbidities, for both people with and without SMI.

## Methods

The Clinical Practice Research Datalink (CPRD) is a large primary care database of anonymised longitudinal medical records which contains detailed information on diagnoses, referrals, prescribed treatments and test results. The version we analysed has been described in detail elsewhere.^18^ We used all available data from 627 practices to extract diagnoses information and aggregated it in 12 yearly bins, from 1 April 2000 to 31 March 2012.

### Generating a code list for SMI and other conditions

We used Read codes to identify the presence of SMI. First, we identified relevant keywords (or key-stubs) and codes, for example, ‘paranoi’ and E1*, covering the mental health domain (see online appendix table A1). Next, the CPRD was searched for codes that matched the list in either the code or the description field. Finally, the matched code list was reviewed by clinical experts, and a final conservative list of codes was agreed. SMI was defined as: schizophrenia, affective disorder (divided into bipolar or unspecified affective disorder) or other types of psychoses, consistent with the inclusion criteria for SMI registers in primary care general practice in the UK as part of the Quality and Outcomes Framework (QOF) financial incentive scheme. The QOF was introduced in 2004 and links GP's pay with achievement of targets set across a range of chronic conditions.^19^

The research team selected 16 conditions from an extended list, based on previous work, incentivisation under the QOF and a review of existing literature. The QOF is relevant since quality of recording is excellent for its domains and we included all except depression (because of coding complexities and potential overlap with SMI) and obesity. The conditions were: hypertension, diabetes (type I and II), asthma, hypothyroidism, osteoarthritis, chronic kidney disease (CKD), learning disability, coronary heart disease, epilepsy, chronic obstructive pulmonary disease (COPD), cancer, stroke, heart failure, rheumatoid arthritis, dementia and psoriasis. These were discussed and agreed a priori by all authors, and codes associated with these conditions were obtained through a similar approach to the one used for patients with SMI and were mainly developed for previous work.^20^ All the code lists we used, as well as the SMI categorisation, are available from http://www.clinicalcodes.org,^21^ while more details on the Read code selection process in this SMI context have been provided elsewhere.^22^

Within each year, all patients registered with a CPRD practice for the whole year and aged 18 or over were eligible for inclusion. The final SMI Read code list was used to identify cases with schizophrenia, affective psychoses (bipolar disorder or other unspecified affective psychosis) and other types of psychosis, in line with the diagnoses used when compiling primary care QOF SMI registers.^23^ If an individual received more than one SMI diagnosis over the study period, we used the last available diagnosis to retrospectively ‘correct’ the original diagnosis (ie, we assumed that the latest diagnosis was the correct one). Within each year, each SMI case was then matched on age, sex and general practice to five randomly selected patients not associated with SMI up until that time point.

### Other sociodemographic characteristics

Deprivation was measured using the 2007 Index of Multiple Deprivation (IMD) score in England,^24^ applied to the practice postcode. Analogous deprivation indexes were used for Welsh, Scottish and NI practices. One of five deprivation quintiles, based on the deprivation distribution within each country, was assigned to each individual in the sample.

### Medications

We reported the number of individuals with one or more prescriptions within a year for each of the following medications: antipsychotic drugs (first, second generation and depot injections), tricyclic antidepressants (TCAs), selective serotonin reuptake inhibitors (SSRIs) and other antidepressants.

### Analysis

SMI prevalence rates were calculated overall, by deprivation quintile and by practice region, across the study period. We report patient characteristics for both SMI and control cases, including prevalence rates for the investigated comorbidities. Finally, we created detailed comorbidity mapping tables for both groups, a common practice when investigating disease clusters,^12^ ^25^ to identify relevant patterns of comorbidity in SMI cases and investigate how they differed to what is observed for controls. Prevalence rates were calculated annually over the study period (2000/2001–2011/2012), although we focused on the last financial year to investigate if the comorbidity patterns differed by SMI diagnosis type (schizophrenia, bipolar disorder, affective disorder and other types of psychosis). We also calculated comorbidity maps for combinations of comorbidities, for both SMI and controls, aggregated over 5 years (2007/2008–2011/2012).

## Results

The number of practices included in the sample ranged from 434 practices in 2000/2001 to 569 in 2006/2007 (table 1; online appendix figure A1). Numbers of individuals with SMI rose year on year from 19 658 in 2000/2001 (434 practices) to a high of 33 117 in 2009/2010 (556 practices) and declined in subsequent years (table 1).

**Table 1:** Characteristics of the SMI population and the matched controls, over time

	2000/2001	2001/2002	2002/2003	2003/2004	2004/2005	2005/2006	2006/2007	2007/2008	2008/2009	2009/2010	2010/2011	2011/2012
Counts (total)												
Number of practices	434	472	503	532	553	566	569	565	565	556	534	499
Number of individuals	3 805 086	4 199 071	4 534 974	4 843 511	5 071 047	5 214 673	5 321 351	5 369 370	5 449 547	5 432 224	5 301 520	5 069 748
SMI	19 658	22 039	24 740	26 969	29 040	30 286	31 267	32 175	32 666	33 117	32 787	31 807
Controls	98 290	110 195	123 700	134 845	145 200	151 430	156 335	160 875	163 330	165 585	163 935	159 035
Counts (most affluent quintile)												
Number of practices	69	74	82	90	92	96	96	96	96	96	94	87
Number of individuals	707 541	756 150	832 676	910 936	952 045	989 010	1 006 610	1 015 637	1 038 967	1 057 164	1 050 114	1 000 798
SMI	3103	3345	3769	4168	4506	4753	4795	4928	5002	5131	5193	4956
Controls	15 515	16 725	18 845	20 840	22 530	23 765	23 975	24 640	25 010	25 655	25 965	24 780
Percentage of SMI most affluent quintile	0.16	0.15	0.15	0.15	0.16	0.16	0.15	0.15	0.15	0.15	0.16	0.16
Counts (most deprived quintile)												
Number of practices	95	99	101	105	109	110	110	108	107	106	102	97
Number of individuals	783 582	842 103	871 663	902 512	930 570	938 429	940 953	927 943	939 433	928 240	903 084	860 807
SMI	4668	5130	5639	6009	6363	6613	6795	6918	6945	7005	6964	6809
Controls	23 340	25 650	28 195	30 045	31 815	33 065	33 975	34 590	34 725	35 025	34 820	34 045
Percentage of SMI most deprived quintile	0.24	0.23	0.23	0.22	0.22	0.22	0.22	0.22	0.21	0.21	0.21	0.21
Percentage of male												
SMI	45.42	46.18	47.10	47.75	48.02	48.42	48.49	48.89	48.63	48.86	48.82	48.93
Controls	45.42	46.18	47.10	47.75	48.02	48.42	48.49	48.89	48.63	48.86	48.82	48.93
Mean age (SD)												
SMI	51.6 (17.6)	51.4 (17.5)	51.2 (17.5)	50.9 (17.4)	50.8 (17.2)	50.8 (17.1)	50.8 (17.0)	50.9 (16.9)	51.1 (16.8)	51.2 (16.8)	51.4 (16.8)	51.6 (16.7)
Control	51.6 (17.6)	51.4 (17.5)	51.2 (17.5)	50.9 (17.4)	50.8 (17.2)	50.8 (17.1)	50.8 (17.0)	50.9 (16.9)	51.1 (16.8)	51.2 (16.8)	51.4 (16.8)	51.6 (16.7)
*Annual prevalence rates of comorbidities and mean number of conditions (in addition to SMI) in all patients diagnosed with SMI and matched controls*												
Hypertension												
SMI	12.20	12.70	13.14	14.22	14.92	15.73	16.35	16.96	17.45	17.80	17.99	18.29
Controls	14.19	14.77	14.95	15.42	16.10	16.34	16.48	16.46	16.58	16.35	16.24	16.11
Diabetes (type I and II)												
SMI	5.29	5.79	6.07	6.48	7.18	7.72	8.34	8.79	9.33	9.82	10.41	11.11
Controls	3.53	3.77	3.85	4.14	4.33	4.49	4.70	4.90	5.05	5.16	5.36	5.55
Asthma												
SMI	5.80	6.14	6.48	7.06	7.29	7.54	7.67	7.82	8.20	8.29	8.18	7.88
Controls	5.44	5.48	5.74	5.75	5.93	5.98	5.88	6.01	5.98	6.03	5.85	5.47
Hypothyroidism												
SMI	5.51	5.75	6.23	6.84	7.45	8.05	8.32	8.56	8.87	9.00	9.06	9.18
Controls	2.96	3.20	3.19	3.54	3.78	3.96	4.01	4.05	4.14	4.25	4.19	4.24
Osteoarthritis												
SMI	8.98	9.05	9.21	9.44	9.60	9.80	9.80	10.05	10.41	10.38	10.71	10.79
Controls	9.36	9.42	9.20	9.24	9.28	9.51	9.53	9.44	9.58	9.61	9.63	9.50
Chronic kidney disease												
SMI	0.28	0.32	0.45	0.55	0.67	0.96	5.48	7.02	7.53	7.88	8.01	8.24
Controls	0.26	0.29	0.30	0.36	0.44	0.57	3.19	3.89	4.14	4.27	4.25	4.16
Learning disability												
SMI	1.33	1.31	1.28	1.24	1.29	1.37	1.48	1.46	1.46	1.75	1.89	1.86
Controls	0.13	0.16	0.15	0.15	0.15	0.16	0.18	0.18	0.16	0.22	0.21	0.22
Coronary heart disease												
SMI	5.71	5.47	5.44	5.21	4.97	4.82	4.76	4.63	4.51	4.50	4.47	4.51
Controls	5.61	5.56	5.31	4.97	4.81	4.60	4.43	4.24	4.00	3.85	3.66	3.60
Epilepsy												
SMI	2.03	2.19	2.17	2.40	2.54	2.60	2.56	2.61	2.55	2.59	2.66	2.58
Controls	0.70	0.76	0.75	0.72	0.82	0.75	0.77	0.75	0.76	0.70	0.71	0.70
COPD												
SMI	1.89	2.01	2.05	1.94	2.10	2.26	2.49	2.74	2.94	3.12	3.20	3.46
Controls	1.36	1.40	1.47	1.43	1.56	1.60	1.60	1.66	1.72	1.72	1.81	1.80
Cancer												
SMI	2.76	2.77	2.93	3.07	3.12	3.22	3.38	3.46	3.50	3.65	3.83	4.11
Controls	2.70	2.78	2.74	2.81	2.87	2.96	3.09	3.06	3.14	3.21	3.39	3.44
Stroke												
SMI	3.64	3.69	3.63	3.53	3.47	3.52	3.45	3.55	3.52	3.59	3.75	3.75
Controls	2.51	2.48	2.42	2.24	2.21	2.09	2.15	2.08	2.09	1.98	1.97	2.07
Heart failure												
SMI	2.28	2.05	2.07	1.99	1.86	1.74	1.55	1.44	1.34	1.31	1.37	1.41
Controls	1.64	1.55	1.44	1.30	1.15	1.06	0.97	0.90	0.88	0.79	0.77	0.79
Rheumatoid arthritis												
SMI	0.92	0.89	0.84	0.86	0.91	0.88	0.89	0.92	0.90	0.95	0.99	1.04
Controls	0.91	0.97	0.94	0.99	0.95	0.95	0.97	0.98	0.92	0.91	0.97	0.95
Dementia												
SMI	2.26	2.23	2.22	2.18	2.24	2.26	2.40	2.40	2.31	2.50	2.63	2.64
Controls	0.49	0.46	0.45	0.44	0.47	0.49	0.49	0.51	0.53	0.55	0.56	0.57
Psoriasis												
SMI	3.02	3.13	3.21	3.28	3.46	3.57	3.74	3.85	3.98	4.07	4.22	4.34
Controls	2.62	2.66	2.81	2.74	2.86	2.90	3.02	3.10	3.15	3.23	3.26	3.40
Mean number of conditions (SD)												
SMI	0.6 (1.0)	0.7 (1.0)	0.7 (1.0)	0.7 (1.1)	0.7 (1.1)	0.8 (1.1)	0.8 (1.2)	0.9 (1.2)	0.9 (1.3)	0.9 (1.3)	0.9 (1.3)	1.0 (1.3)
Control	0.5 (0.9)	0.6 (0.9)	0.6 (1.0)	0.6 (1.0)	0.6 (1.0)	0.6 (1.0)	0.6 (1.1)	0.6 (1.1)	0.6 (1.1)	0.6 (1.1)	0.6 (1.1)	0.6 (1.1)

COPD, chronic obstructive pulmonary disease; SMI, severe mental illness.

**Figure 1:**
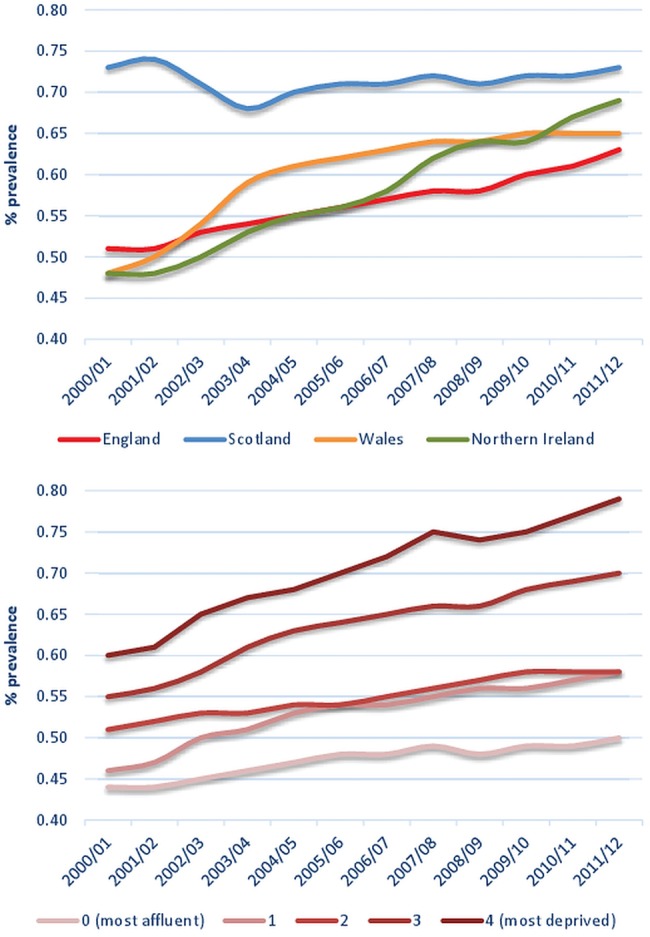
Prevalence of severe mental illness by UK country (top) and deprivation quintile (bottom), over time.

In Scotland, in 2011/2012, the annual prevalence of SMI was 0.73% compared with 0.69% in NI, 0.65% in Wales and 0.63% in England (table 2 and figure 1). The prevalence rate of SMI in Scotland did not increase overall during the study period, though in the most deprived quintile, some increases were observed. In contrast, rates increased in England, Wales and especially in NI (from 0.48 in 2000/2001 to 0.69 in 2011/2012). With the exception of the South West, the annual prevalence rate of SMI increased across all English regions over the study period (table 2). The greatest increases were observed in the North East, North West and the East Midlands. SMI prevalence was the highest in areas in the two highest deprivation quintiles (table 2 and figure 1). Changes over time were more evident in the most deprived quintile in NI with rates more than doubling over the 12-year period (0.49 in 2000/2001 to 1.16 in 2011/2012), with a great rise in 2007/2008 (table 2). The mean number of years since the first SMI diagnosis increased from 11.7 (SD 11.5) in 2000/2001 to 13.2 (SD 11.8) in 2011/2012 and the mean years since the final diagnosis increased from 9.1 (SD 11.3) in 2000/2001 to 11.5 (SD 11.0) in 2011/2012 (table 3).

**Table 2:** Annual severe mental illness (SMI) prevalence rates (all patients diagnosed with SMI) by geographical location and area deprivation quintile, over time

	2000/01	2001/02	2002/03	2003/04	2004/05	2005/06	2006/07	2007/08	2008/09	2009/10	2010/11	2011/12
UK												
Overall	0.52	0.52	0.55	0.56	0.57	0.58	0.59	0.60	0.60	0.61	0.62	0.63
0 (most affluent)	0.44	0.44	0.45	0.46	0.47	0.48	0.48	0.49	0.48	0.49	0.49	0.50
1	0.46	0.47	0.50	0.51	0.53	0.54	0.54	0.55	0.56	0.56	0.57	0.58
2	0.51	0.52	0.53	0.53	0.54	0.54	0.55	0.56	0.57	0.58	0.58	0.58
3	0.55	0.56	0.58	0.61	0.63	0.64	0.65	0.66	0.66	0.68	0.69	0.70
4 (most deprived)	0.60	0.61	0.65	0.67	0.68	0.70	0.72	0.75	0.74	0.75	0.77	0.79
*Country*												
England*												
Overall	0.51	0.51	0.53	0.54	0.55	0.56	0.57	0.58	0.58	0.60	0.61	0.63
Most affluent	0.44	0.43	0.45	0.45	0.46	0.46	0.45	0.46	0.45	0.46	0.47	0.47
Most deprived	0.62	0.63	0.66	0.66	0.67	0.69	0.71	0.73	0.72	0.75	0.77	0.80
Northern Ireland†												
Overall	0.48	0.48	0.50	0.53	0.55	0.56	0.58	0.62	0.64	0.64	0.67	0.69
Most affluent	0.41	0.44	0.44	0.48	0.48	0.51	0.53	0.54	0.56	0.56	0.59	0.60
Most deprived	0.49	0.49	0.52	0.58	0.66	0.71	0.74	1.03	1.04	1.09	1.09	1.16
Scotland‡												
Overall	0.73	0.74	0.71	0.68	0.70	0.71	0.71	0.72	0.71	0.72	0.72	0.73
Most affluent	0.63	0.66	0.56	0.53	0.56	0.56	0.55	0.56	0.54	0.55	0.55	0.57
Most deprived	0.77	0.80	0.89	0.94	0.95	0.97	0.97	0.97	0.98	0.98	0.98	1.00
Wales§												
Overall	0.48	0.50	0.54	0.59	0.61	0.62	0.63	0.64	0.64	0.65	0.65	0.65
Most affluent	0.49	0.5	0.53	0.56	0.59	0.61	0.61	0.62	0.6	0.6	0.63	0.59
Most deprived	0.43	0.48	0.53	0.56	0.58	0.59	0.61	0.61	0.61	0.64	0.65	0.66
English regions												
North East	0.49	0.51	0.53	0.53	0.54	0.55	0.57	0.64	0.62	0.64	0.67	0.69
North West	0.54	0.56	0.60	0.62	0.65	0.67	0.67	0.68	0.68	0.69	0.72	0.74
Yorkshire	0.67	0.60	0.59	0.60	0.60	0.59	0.59	0.60	0.65	0.68	0.67	0.75
East-Midlands	0.45	0.46	0.49	0.51	0.54	0.54	0.56	0.53	0.53	0.57	0.57	0.65
West-Midlands	0.46	0.47	0.49	0.50	0.50	0.50	0.51	0.52	0.51	0.51	0.52	0.53
East of England	0.50	0.52	0.53	0.53	0.54	0.54	0.55	0.57	0.58	0.58	0.61	0.63
South West	0.55	0.54	0.54	0.53	0.52	0.51	0.51	0.49	0.50	0.52	0.52	0.52
South Central	0.45	0.45	0.47	0.48	0.49	0.50	0.50	0.52	0.51	0.51	0.51	0.51
London	0.58	0.60	0.64	0.65	0.66	0.68	0.69	0.71	0.71	0.73	0.73	0.73
South East	0.40	0.41	0.43	0.45	0.48	0.50	0.51	0.52	0.54	0.54	0.55	0.55

*Overall number of practices within each quintile from most affluent to most deprived: 73, 103, 103, 111 and 96.

†Overall number of practices within each quintile from most affluent to most deprived: 5, 5, 2, 7 and 3.

‡Overall number of practices within each quintile from most affluent to most deprived: 14, 11, 18, 11 and 14.

§Overall number of practices within each quintile from most affluent to most deprived: 7, 6, 11, 16 and 11.

**Table 3:** Prevalence, number and gender for all individuals with severe mental illness (SMI) diagnosis*, mean years with first and latest SMI diagnosis and prescribed medications†, over time

	2000/2001	2001/2002	2002/2003	2003/2004	2004/2005	2005/2006	2006/2007	2007/2008	2008/2009	2009/2010	2010/2011	2011/2012
Prevalence of individuals with SMI diagnosis												
Schizophrenia	0.11	0.11	0.12	0.12	0.13	0.13	0.13	0.14	0.13	0.14	0.14	0.14
Bipolar disorder	0.13	0.13	0.14	0.15	0.15	0.16	0.16	0.17	0.17	0.18	0.18	0.19
Unspecified/other affective psychosis	0.06	0.06	0.06	0.06	0.06	0.06	0.06	0.07	0.07	0.07	0.07	0.07
Other types of psychosis	0.18	0.18	0.19	0.2	0.2	0.2	0.2	0.21	0.21	0.21	0.22	0.23
Number of individuals with SMI diagnosis (not exclusively)												
Schizophrenia	4098	4674	5268	5842	6339	6727	7059	7281	7296	7323	7205	7029
Bipolar disorder	4906	5613	6352	7083	7784	8259	8701	9055	9332	9715	9727	9496
Unspecified/other affective psychosis	1938	2150	2392	2573	2839	2999	3255	3398	3551	3690	3696	3605
Other SMI	6862	7691	8764	9551	10 226	10 628	10 857	11 226	11 484	11 623	11 665	11 631
Percentage of male individuals with SMI diagnoses												
Schizophrenia	54.86	55.63	57.52	58.37	58.98	59.86	60.41	60.86	60.68	61.35	61.5	61.79
Bipolar disorder	39.52	39.66	39.97	39.93	39.92	39.77	39.46	39.72	39.71	39.66	39.8	39.49
Unspecified/other affective psychosis	32.04	33.26	34.36	35.29	35.51	35.45	36.65	37.7	37.37	37.32	37.28	37.14
Other types of psychosis	48.32	49.25	49.55	50.38	50.76	51.4	51.39	51.88	51.68	52.34	52.2	52.51
Mean number of years with SMI diagnosis (SD)												
First SMI diagnosis	11.7 (11.5)	11.8 (11.6)	11.8 (11.6)	11.8 (11.6)	11.8 (11.6)	12.1 (11.7)	12.2 (11.7)	12.3 (11.6)	12.6 (11.7)	12.8 (11.7)	13.0 (11.7)	13.2 (11.8)
Latest SMI diagnosis	9.1 (11.3)	9.2 (11.3)	9.4 (11.2)	9.5 (11.2)	9.6 (11.1)	9.9 (11.1)	10.1 (11.0)	10.3 (10.9)	10.6 (10.9)	10.9 (10.9)	11.3 (11.0)	11.5 (11.0)
Antipsychotics‡												
First generation (conventional)	20.26	20.11	17.64	15.31	14.79	13.84	12.71	11.94	11.03	10.12	9.39	8.78
Second generation (atypical)	18.1	23.03	27.12	30.68	33.33	35.77	37.75	38.8	39.82	40.57	41.77	43.02
Depot	5.95	5.65	5.15	4.69	4.45	4.13	3.83	3.65	3.5	3.5	3.32	3.23
Antidepressants‡												
Tricyclic	14.03	12.94	12.23	11.81	11.17	10.32	9.81	9.4	9.28	9.19	9.32	9.21
Selective serotonin reuptake inhibitors	18.13	19.65	20.27	20.41	20.92	21.16	22.58	22.95	23.8	24.38	25.23	25.75
Other antidepressants§	6.87	8.7	10.05	11.18	12.21	12.3	12.67	13.17	13.31	13.83	14.5	14.96

*In instances where an individual received more than one SMI diagnostic subcategory diagnoses during the study period, the patient was assigned to the last diagnostic category received, since it was likely to be based on a greater knowledge of the individual’s clinical history.

†Patients were considered to be associated with a medication group if they received at least one prescription of a relevant drug within the respective year.

‡Organised by chapters in British National Formulary (BNF) 67 (March 2014).

§Not listed in BNF 67 (March 2014).

The prevalence rates for all of the diagnostic categories (bipolar disorder: 0.13–0.19; other SMI: 0.18–0.23; and schizophrenia: 0.11–0.14) increased over the study period with the least increase in unspecified/other affective psychosis (0.06–0.07).

### Medication: antipsychotic medication and antidepressants

The number of individuals with one or more prescription for first generation antipsychotic medications has steadily declined over the 12 years (from 20.26% to 9.78%), whereas second generation antipsychotic medications have steadily increased (from 18.1% to 43%). Decreases in depot injections were observed over time as shown in table 3. An increase over the time period was observed for SSRIs (from 18.1% to 25.75%), whereas a decrease was observed for TCAs (from 14.03% to 9.21%).

### Prevalence rates of comorbidities

Annual prevalence rates varied over the 12-year period for those with and without SMI (table 1). There appeared to be a greater increase in the mean number of additional conditions in the SMI population over the study period (0.6 in 2000/2001 to 1.0 in 2011/2012) compared with control cases (0.5 in 2000/2001 to 0.6 in 2011/2012; table 1).

Over time, prevalence rates for comorbidities generally increased for both groups but the increases were greater for the SMI group. For example, hypertension prevalence increased from 12.2 to 18.3 between 2000/2001 and 2011/2012 for the SMI group, but for the control group, the increase was smaller from 14.2 to 16.3 over the same time period. There were some notable exceptions: the rates for coronary heart disease and heart failure fell for both groups, and the rates for stroke remained relatively stable for the SMI group but fell for the controls.

**Figure 2:**
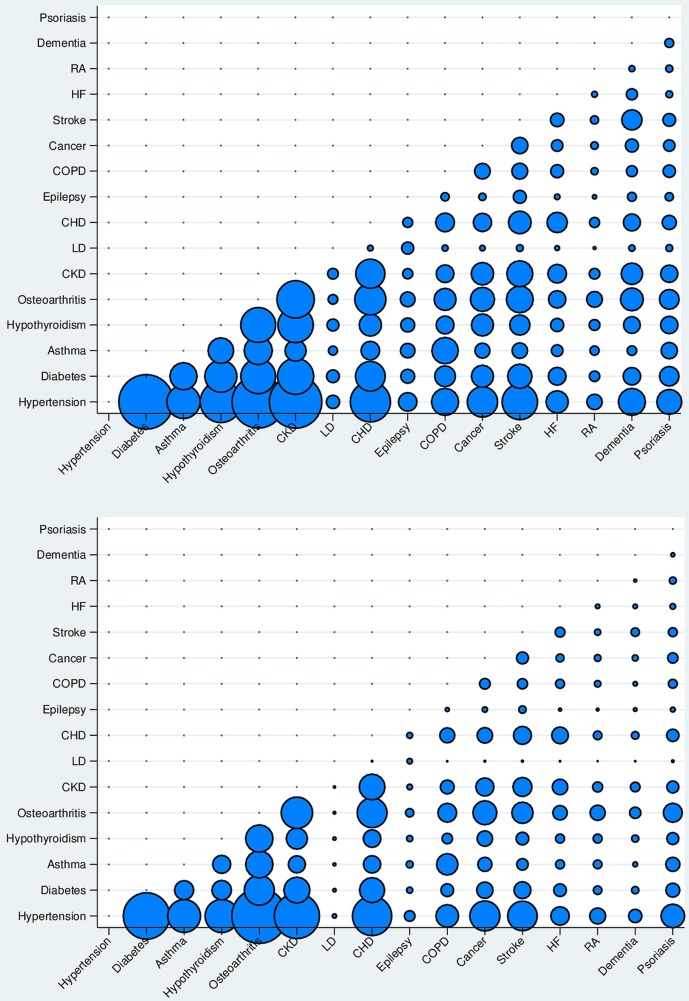
(top) Patterns of comorbidities for SMI (top) and control patients (bottom) over 5 years (2007/2008–2011/2012); percentage of patients with two specific conditions (see also table A3). CHD, coronary heart disease; CKD, chronic kidney disease; COPD, chronic obstructive pulmonary disease; HF, heart failure; LD, learning disability; RA, rheumatoid arthritis; SMI, severe mental illness.

Although percentage of patients with two specific conditions differed, comorbidity combinations were similar for the patients with SMI and matched controls. The most common comorbidity combination (percentage of patients with 2 conditions) was diabetes and hypertension and was experienced by 4.4% of patients with SMI (figure 2; online appendix tables A2 and A3). The percentages of people with one of the conditions who also have other conditions were higher for the SMI group for most conditions (see online appendix table A4 and figure A2). However, higher rates of hypertension were observed in the controls with a diagnosis of diabetes, asthma, hypothyroidism, osteoarthritis, CKD, COPD and heart failure.

A variable pattern of comorbidity was observed across the different SMI subgroups and areas of high and low deprivation (see online appendix tables A5–7). When we focused on 2011/2012 data and compared SMI and control cases within each SMI diagnosis subgroup, we observed differences in prevalence rates for almost all conditions in all groups (see online appendix table A5). We noted large differences between the prevalence rates of a number of conditions such as diabetes mellitus, hypothyroidism, CKD for people with SMI and those without SMI. There were also differences between SMI diagnoses groups (schizophrenia, bipolar disorder, affective disorder and other types of psychosis). For example, the difference between prevalence rates of people with schizophrenia and controls who also had a diabetes diagnosis (13.42 compared with 6.01) was larger than the difference between the other three SMI diagnoses groups and controls. The difference between prevalence rates of people with bipolar disorder and controls who also had a hypothyroidism diagnosis (12.77 compared with 4.58) and a CKD diagnosis (10.41 compared with 3.9) than the difference between the other three SMI diagnoses groups and controls. In addition, for diabetes mellitus, asthma, CHD, COPD, learning disability, osteoarthritis and epilepsy, we observed higher prevalence rates in people with SMI in the most deprived quintile (see online appendix table A6). For hypothyroidism, CKD, psoriasis, cancer, stroke and dementia, prevalence rates were higher in the most affluent quintile (see online appendix table A7).

## Discussion

### Findings

This is the first large longitudinal study to explore the prevalence of SMI and comorbidity in the context of deprivation. We identified a number of key findings: First, we found that the prevalence of SMI, in the UK, increased over the 12-year period from 2000 to 2012. The increase was consistent across all diagnosis subgroups but highest in bipolar disorder and other SMI. Increases were highest in areas of high social deprivation. Second, the difference in rates across England, NI, Wales and Scotland has narrowed overtime. Third, the age at which people received their diagnosis lowered over the 12 years (age stayed constant while the mean number of years since the first SMI diagnosis increased). Fourth, we observed an increase in the average number of reported comorbidities for the SMI group. Some conditions increased at a higher rate for those with a SMI diagnosis: COPD, diabetes, hypothyroidism and asthma. Whereas the rates for hypertension, CKD and stroke increased in the SMI group in contrast to control cases which showed a decrease. This also coincides with yearly increase in the proportion of people with SMI with one or more prescription of atypical antipsychotic medications. Finally, a variable pattern of comorbidity was observed across the different SMI subgroups and areas of high and low deprivation.

### Comparison with previous research

Our study is one of the latest in a growing number of studies that have used electronic patient health records to examine epidemiological data and multimorbidity associated with people with SMI.^2^ ^13^ ^15^ ^26^ The prevalence rate of SMI observed in the final study year (2011/2012) is lower than reported by the English QOF (0.63 compared with 0.87),^18^ and previous estimates.^27^ Our 2007 rates for Scotland (0.71/0.72) were similar to those recently reported (0.70).^13^ Differences are common in studies which use different Read code lists to define SMI. For example, the lower rates in our study may be partly attributed to the exclusion of drug/alcohol-induced psychoses, organic psychoses, dementia, unipolar depression, personality disorders and psychotic disorders in childhood/adolescence. The publication of our Read code lists, through http://www.clinicalcodes.org, will facilitate future research in this field and the comparison of rates across future studies.

It is not possible within this study to explain the reasons for the increase in SMI rates over time. Our results indicate that people are getting diagnosed earlier. However, further research is needed to explore the factors that may contribute to these observed increases.

Although there have been recent studies providing estimates of prevalence, they examined a more limited set of comorbidities,^2^ ^16^ they did not have UK coverage or provide estimates for all SMI subgroups.^13^ ^15^ To our knowledge, there are currently no other comparable studies that have examined rates of comorbidities over such a long period of time. Some of our annual findings are consistent with other studies. The 2011/2012 prevalence rates for eight conditions (hypertension, asthma, hypothyroidism, CKD, epilepsy, cancer, stroke, psoriasis) were higher than reported in two previous studies.^13^ ^15^ Other UK studies have not reported dementia prevalence rates for individuals with SMI; our 2011/2012 estimates were higher than the general population (2.64 compared with 0.57). For the same time period, Barnett *et al*^12^ reported 0.7 with dementia in the general population comparing to 0.49 in our control population for the same year (2007). When we compared our prevalence rates for the controls with this study for comparable conditions and year, most rates were within a ±0.5% range. The rates in our study were higher for diabetes (16.48 compared with 13.4), CKD (3.19 compared with 1.9), psoriasis (3.02 compared with 0.7) but lower for COPD (1.6 compared with 3.2). Although there is a growing literature on analysis of clustering conditions,^25^ it was not possible to compare our findings on the most common comorbidity combinations (percentage of patients with 2 conditions) with any of the SMI studies. Although one other study examined combinations of comorbidities, percentages were not reported.^15^

Others have also shown that the presence of a mental health disorder (not just SMI) increased as the number of physical morbidities and is much more likely in more deprived people.^12^ It is possible that the increased number of certain conditions may be linked with increased prescriptions of antipsychotic medications.^28–30^ Furthermore, the lower annual prevalence levels in early years in some conditions may be due to under-reporting in primary care. It is possible that initiatives such as the QOF, the Commissioning for Quality and Innovation (CQUINs) payments framework and the Cardiometabolic Health Resource^31^ may be helping to improve diagnosis and thus leading to increases in prevalence rates. This may indicate a general underdiagnosis of conditions and may therefore be an underestimation of the prevalence among patients with SMI. Others have noted that studies based on medical records will underestimate multimorbidity because some diseases are undiagnosed, and because they will not identify people who do not consult.^32^ In a separate paper, we show that consultations increased over the 12-year period,^33^ which fits with previous evidence indicating that people who consult more often may have more conditions diagnosed.^34^ Smith *et al* observed a systematic under-recognition which might contribute to the substantial cardiovascular-related morbidity and premature mortality observed in patients with schizophrenia.^12^ Our data would suggest that this may also be the case for other SMI subgroups too.

The provision of good medical care tends to vary inversely with need,^35^ and could account for the higher rates of SMI and comorbidity in areas of higher social deprivation; however, we speculate that better case finding, driven by the QOF, partially accounts for the observed increases in the comorbidity burden for the QOF-incentivised SMI group, not observed in the matched controls.^20^ ^36^

Further research is required to examine the upward trend in many of the conditions and action is urgently required to identify the accurate prevalence rates for people with SMI. The recent recommendation to include mental health experts on all National Institute for Health and Care Excellence (NICE) guideline development groups for physical conditions to ensure that the mental health aspects of conditions are comprehensively considered is timely.^37^ The findings are relevant for NHS strategic and operational plans addressing outcomes related to improving health, reducing health inequalities and parity of esteem.^38^ There is an ambition to achieve a genuine parity of esteem between mental and physical health by 2020, and an expectation that each clinical commissioning group is spending on mental health services in 2015/2016 increases in real terms.^39^ The patterns of comorbidities in the most affluent and most deprived quintiles indicate that the effect of deprivation is worth further exploration in conjunction with the impact on clinical activity. For example, our data may suggest that people with SMI registered at a more affluent practice may be more likely to undergo regular monitoring making the diagnosis of unrelated diseases such as cancer and hypothyroidism more likely. It may also be worth investigating comorbidity interactions as the diagnosis of certain conditions may be made easier by the presence of an SMI diagnosis.^40^

The interaction between mental and physical health problems increases the costs of care which have been shown to be greater than the combined costs of having the individual conditions alone.^41^ Despite this, the level of mental health funding is not commensurate with burden and is lower than other chronic conditions.^42^ Having up to date epidemiological data helps to pinpoint how healthcare providers need to meet the challenge of providing good quality treatment and care to people with SMI.

### Strengths and limitations

To our knowledge, this is the first 12-year longitudinal study to examine prevalence rates of 16 comorbidities for people with SMI compared with a matched control group without SMI. We presented these by deprivation quintile and by type of SMI diagnosis. This study demonstrates how research can use routinely collected healthcare data for this purpose.^43^

Databases which use routinely collected clinical data, such as the CPRD, come with certain limitations. Although the CPRD is representative of the UK in its distribution of practice location deprivation, it tends to recruit larger than average practices,^18^ while the version we analysed only included practices utilising one of three major clinical computer systems available (VISION). Although differences in performance across systems have been observed,^44^ such issues may be less relevant in this setting where we focus on prevalence rates. The main limitation, however, is that this is an observational study and the possibility of unmeasured confounding is present. Controls were matched for age, gender and practice (and since deprivation was measured at the practice level, on deprivation as well). Other important parameters such as obesity, unemployment, ethnicity, smoking status, alcohol or illegal drug use were not extracted as they may be recorded inaccurately or missing. However, the nature of the study was purely descriptive and we do not attempt to quantify associations, rather quantify overall differences in physical comorbidity irrespective of the potential underlying factors (a much stricter matching would be at risk of overcontrolling). Since we did not have specific hypotheses about the variations in prevalence rates across deprivation quintiles or between SMI and control cases, and the potential comparisons are numerous, we refrained from testing for statistically significant differences. Instead we focussed on the size of the prevalence rates and their apparent differences. Furthermore, statistical significance is less meaningful for data sets of this size.^45^ Second, the deprivation scores relate to the area location of the general practice and not of the individual's area of residence and therefore may be a less accurate proxy of the individual level of deprivation.^46^ Third, we could not quantify the severity of mental illness or of the comorbidities we investigated. Fourth, some of our estimates for specific countries and deprivation quintiles, especially for NI, are based only on a very small number of practices. Fifth, accurate identification of people with SMI requires a valid and reliable measurement of diagnosis. However, not only is there is a lack of agreement on precise diagnostic category after a diagnostic assessment,^47^ psychiatric diagnoses are highly variable and changeable over time and people will move between diagnoses as the nature of their illness becomes clearer (or indeed less clear). Finally, studies based on medical records will underestimate multimorbidity because some diseases are undiagnosed, and because they will not identify people who do not consult.^32^

### Conclusions and implications for research and practice

Our findings help our understanding of the prevalence of SMI and physical comorbidities and show rising inequalities in the pattern and number of different comorbidities and will be of interest to the scientific community, policymakers, people with SMI, their carers and professionals. Reducing these health inequalities will require adequately funded vigorous health prevention and promotion to improve the management of comorbidities with more intense action in areas of greater social and economic disadvantage. Exactly how and where this is done requires urgent attention and should be informed by the patterns of comorbidity that are most common for this group. This research provides further evidence for a number of recommendations for the NHS that have recently been made including:

- Commissioners and service providers need to be clear about the responsibilities of primary and secondary care services for monitoring and managing the physical health of people with mental health problems, starting from the beginning of treatment with identified health needs acted on quickly.
- All mental health professionals should receive basic physical health training as part of their mandatory training.
- Rates of people accessing interventions included in the QOF should be in line with predicted prevalence of the illness.^48^ ^49^

The knowledge added in this study about patterns of comorbidities associated with SMI will be helpful in the development of studies aimed at investigating causal associations. Multimorbidity is a possible confounding factor, so will be important for planning intervention trials ensuring that participants with SMI are representative regarding their morbidity burden and patterns of illnesses.
